# Supplementary material for: Dual fragmentation via collision-induced and oxygen attachment dissociations using water and its radicals for C=C position-resolved lipidomics
Source: Commun Chem. 2025 May 13;8:148. doi: 10.1038/s42004-025-01525-y (PMC12075507; doi:10.1038/s42004-025-01525-y)
Supplement: Supplementary file 2 — Supporting information [file 42004_2025_1525_MOESM2_ESM.pdf]

Supporting Information

Dual fragmentation via collision-induced and oxygen attachment dissociations using water and its radicals for C=C position-resolved lipidomics

Hiroaki Takeda[1,2,*], Mami Okamoto[3], Hidenori Takahashi[3], Bujinlkham Buyantogtokh[1], Noriyuki Kishi[2,4], Hideyuki Okano[2,4], Hiroyuki Kamiguchi[2], Hiroshi Tsugawa[1,5,6,*]

1. Department of Biotechnology and Life Science, Tokyo University of Agriculture and Technology, Koganei, Tokyo, Japan
2. RIKEN Center for Brain Science, Wako, Saitama, Japan
3. Shimadzu Corporation, 1 Nishinokyo-Kuwabaracho Nakagyo-ku, Kyoto, Japan
4. Keio Regenerative Medicine Research Center, Kawasaki, Kanagawa, Japan
5. RIKEN Center for Sustainable Resource Science, Yokohama, Kanagawa, Japan
6. RIKEN Center for Integrative Medical Sciences, Yokohama, Kanagawa, Japan

Corresponding Authors

Hiroaki Takeda (H. Takeda: hiroaki.takeda@aist.go.jp)

Hiroshi Tsugawa (H. Tsugawa: htsugawa@go.tuat.ac.jp)

Contents

Supplementary Figure 1–7

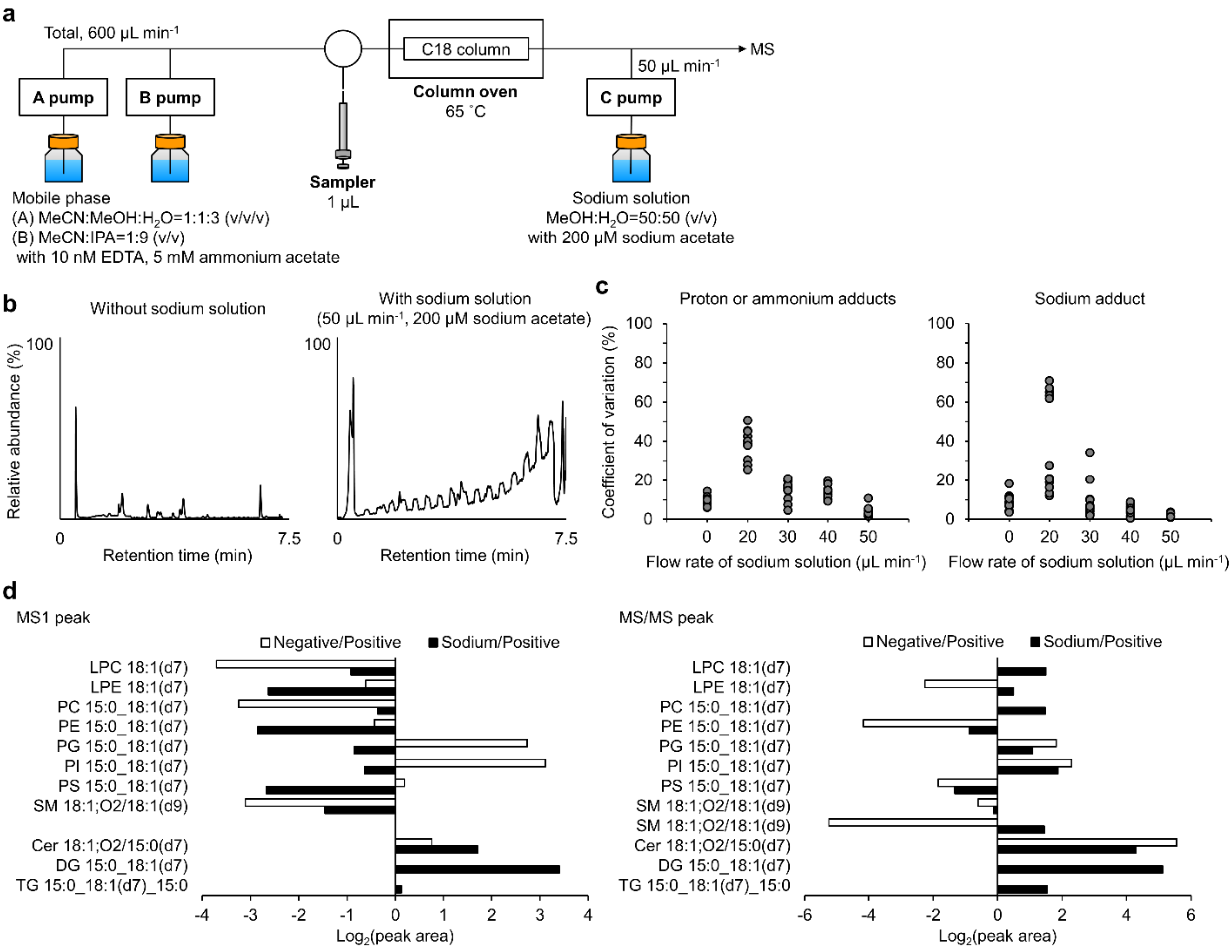

Figure S1. Structural analysis of lipid synthetic standards using sodium adduct. (**a**) Schematic of the LC system for adding the sodium solution. (**b**) Total ion chromatogram of LC systems without or with sodium solution. (**c**) Coefficient of variation at each flow rate of sodium solvent. Each plot shows the synthetic standards in the EquiSPLASH mixture. For all proton, ammonium, and sodium adduct ions, 50 µL min^{-1} of sodium solvent represents high reproducibility (Supplementary Data 1). (**d**) Comparison of the sensitivity for each adduct ion by calculating the MS^1 and MS^2 peak areas. The positive and negative were analyzed using the conventional LC system without sodium solution. The sodium adduct was formed by confluence of the column eluent and sodium solution. The MS^2 peaks were obtained using the product ion scan mode. Two types of SM 18:1;O2/18:1(d9) in the right panel (MS/MS peak) represent the C=C positions derived from the sphingosine base (*m/z* 528.41) and the *N*-acyl chain (*m/z* 633.46), listed in order from top to bottom (Supplementary Data 1).

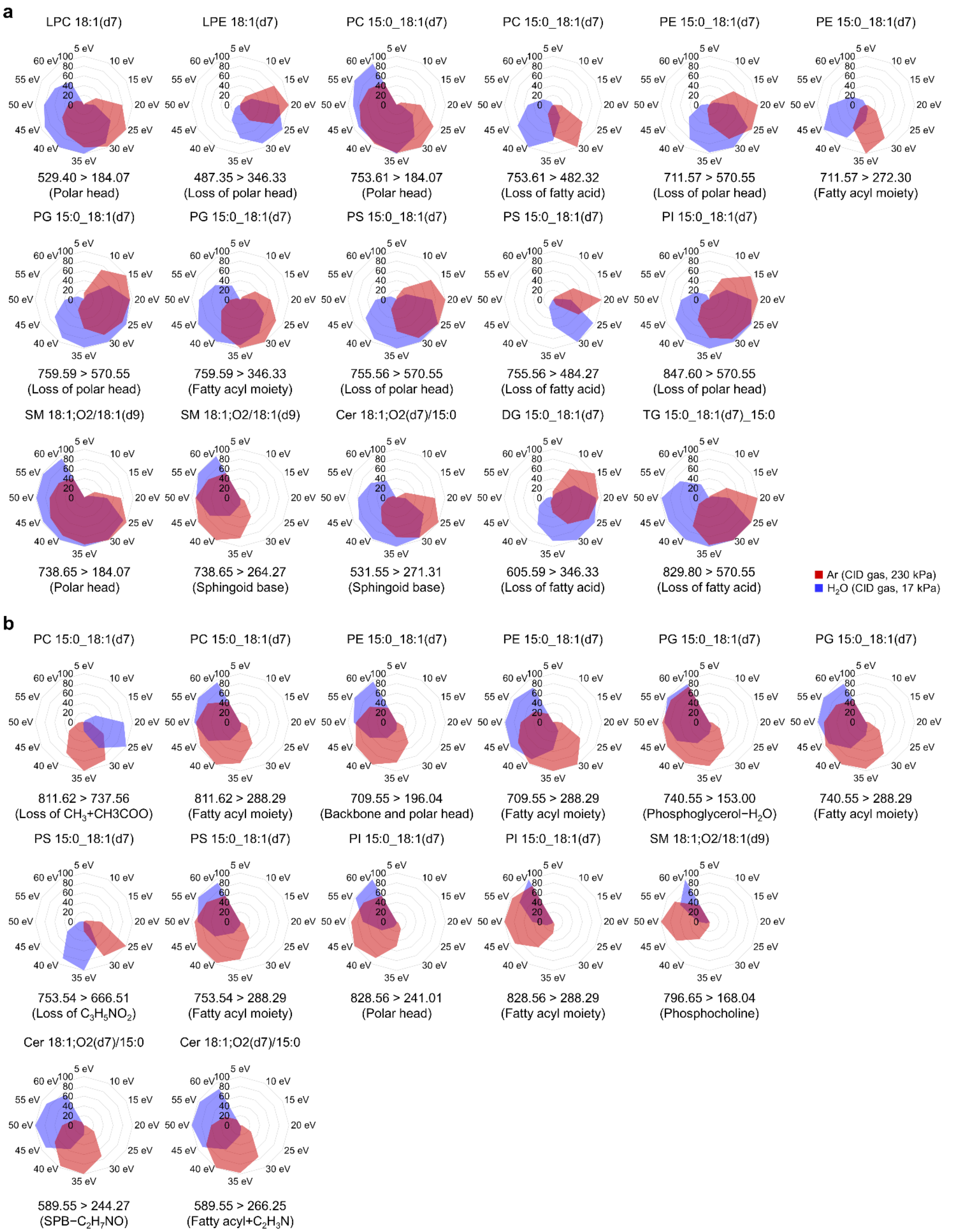

Figure S2. Comparison of fragmentations between Ar gas and H_2O vapor. The area value of MS^2 peak was normalized to the maximum peak detected under the collision energy range of 5–60 eV (Supplementary Data 2). (**a**) Results in positive ion mode. (**b**) Results in negative ion mode.

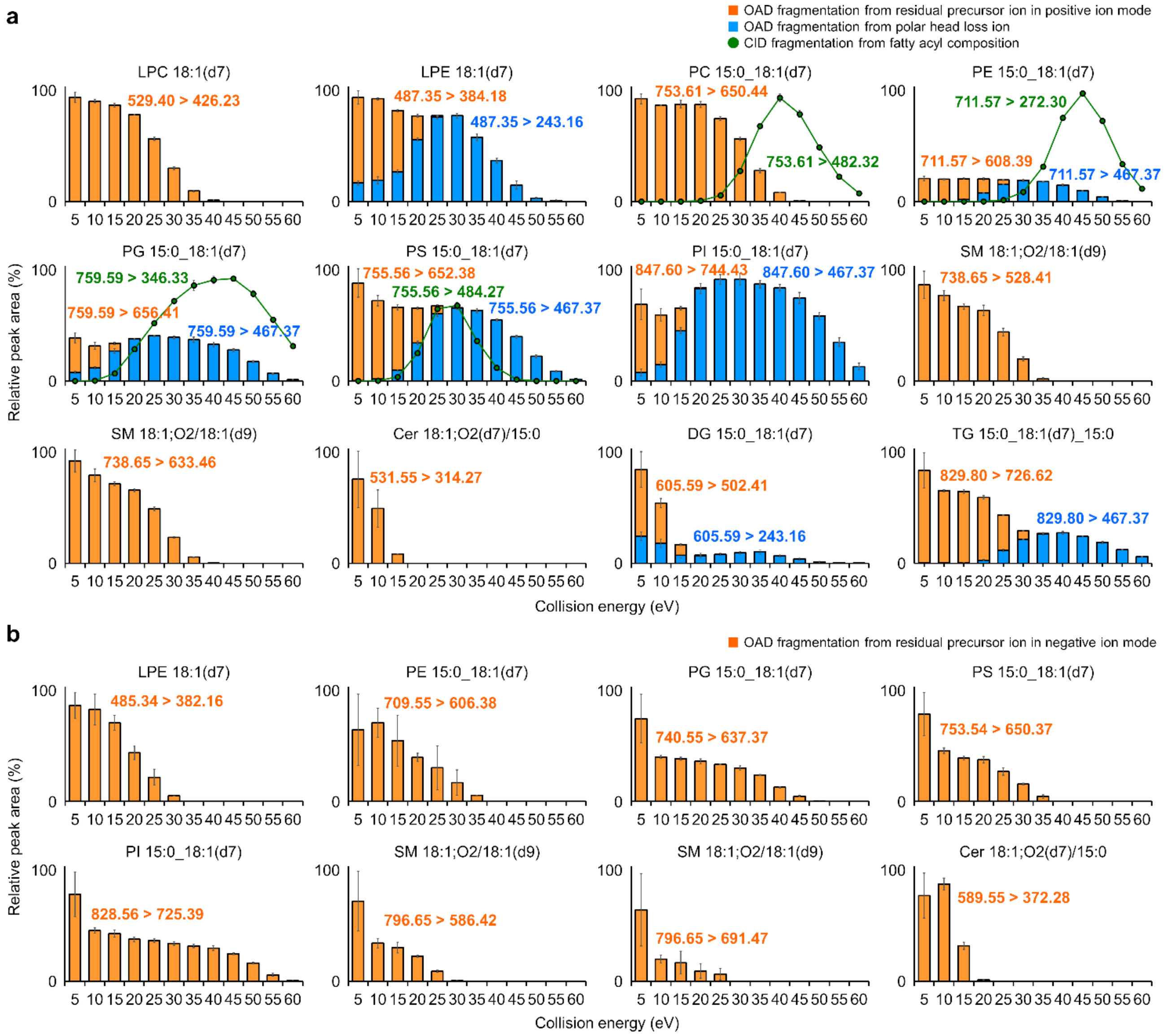

Figure S3. Fragmentation at the C=C position by increasing the collision energy. The orange and blue bars represent the MS^2 peak area of oxidative fragments at the C=C position of residual precursor and polar head loss ions, respectively. The green line plot shows the fragments at the fatty acyl chains, also observed in CID mode using Ar gas. Relative peak areas (%) denote the ion intensities in the product ion spectrum, with the most intense peak set to 100%. Error bars indicate the standard deviations of the four analytical replicates (Supplementary Data 2). (**a**) Results in positive ion mode. (**b**) Results in negative ion mode, excluding CID fragmentation of fatty acid composition, which is about ten times higher than OAD fragmentation to make the visualization clearer.

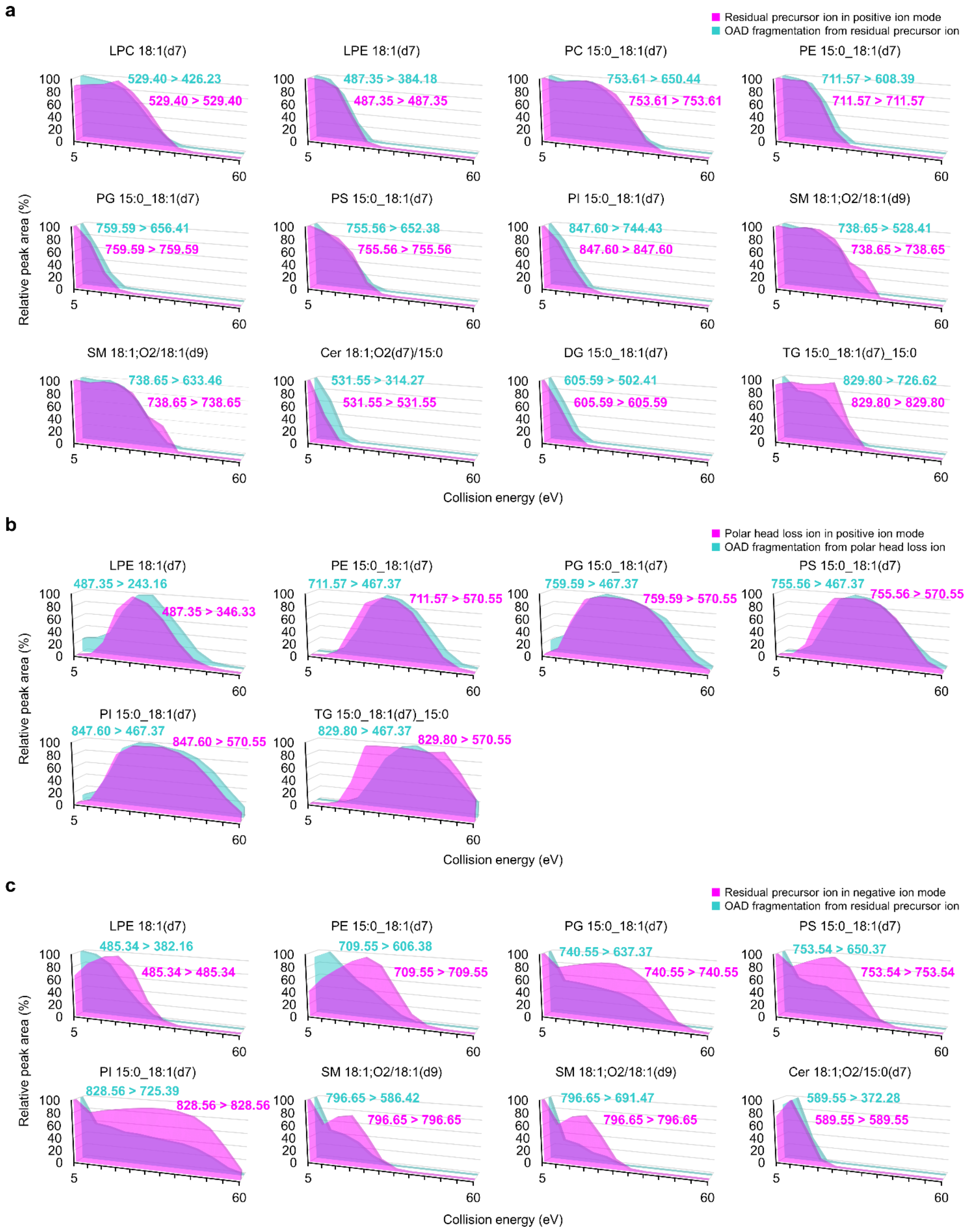

Figure S4. Fragmentation at the C=C position by increasing the collision energy. The MS^2 peak area of fragments is shown at the C=C position from residual precursor or polar head loss ions. Reactants and their OAD fragments at the C=C position are shown in pastel pink and blue, respectively (Supplementary Data

2). Oxidative fragments at the C=C position of residual precursor ions in positive ion mode (**a**), polar head loss ions in positive ion mode (**b**), and residual precursor ions in negative ion mode (**c**) are summarized.

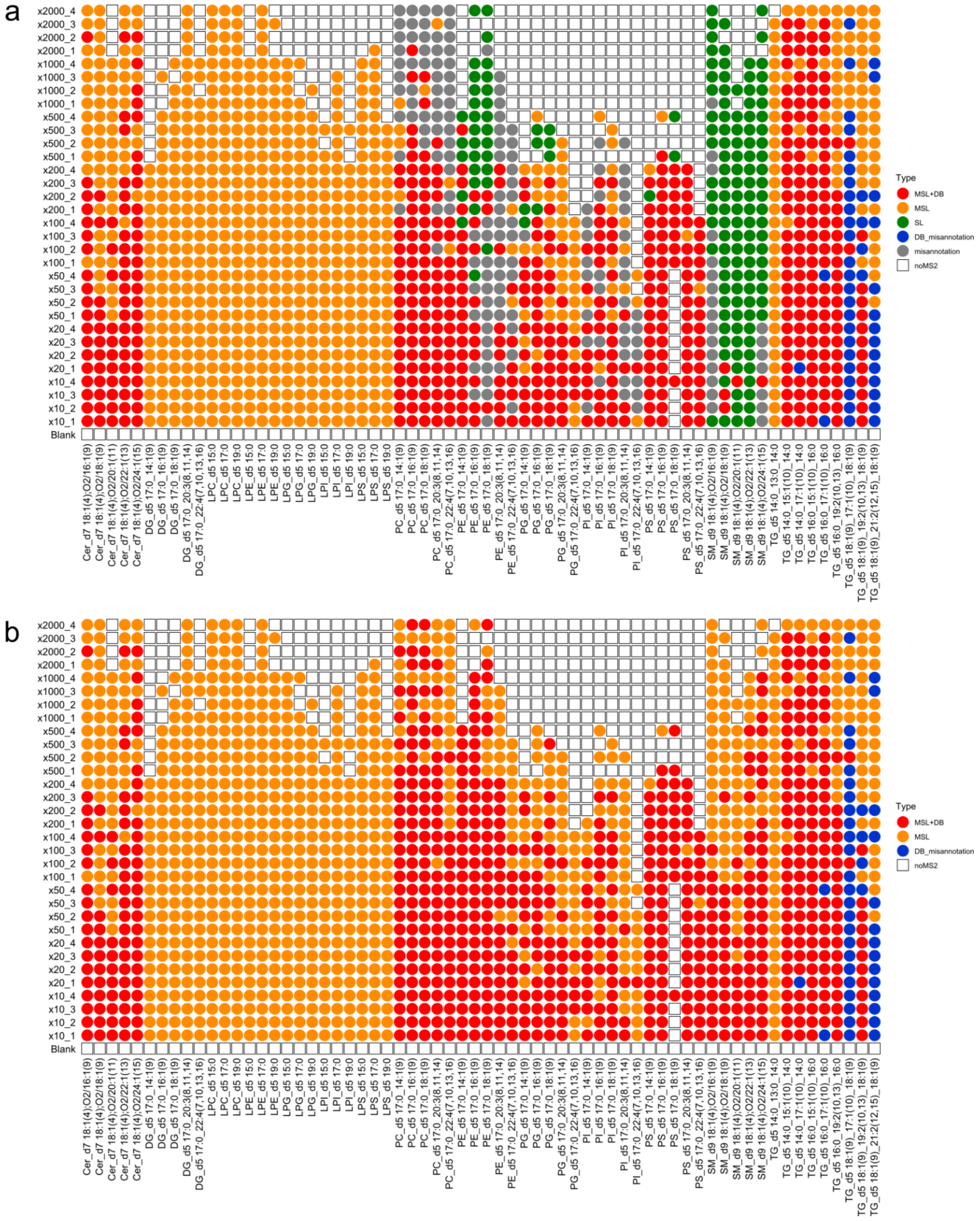
a
b
x2000_4
x2000_3
x2000_2
x2000_1
x1000_4
x1000_3
x1000_2
x1000_1
x500_4
x500_3
x500_2
x500_1
x200_4
x200_3
x200_2
x200_1
x100_4
x100_3
x100_2
x100_1
x50_4
x50_3
x50_2
x50_1
x20_4
x20_3
x20_2
x20_1
x10_4
x10_3
x10_2
x10_1
Blank
Type
MSL+DB
MSL
SL
DB_misannotation
misannotation
noMS2

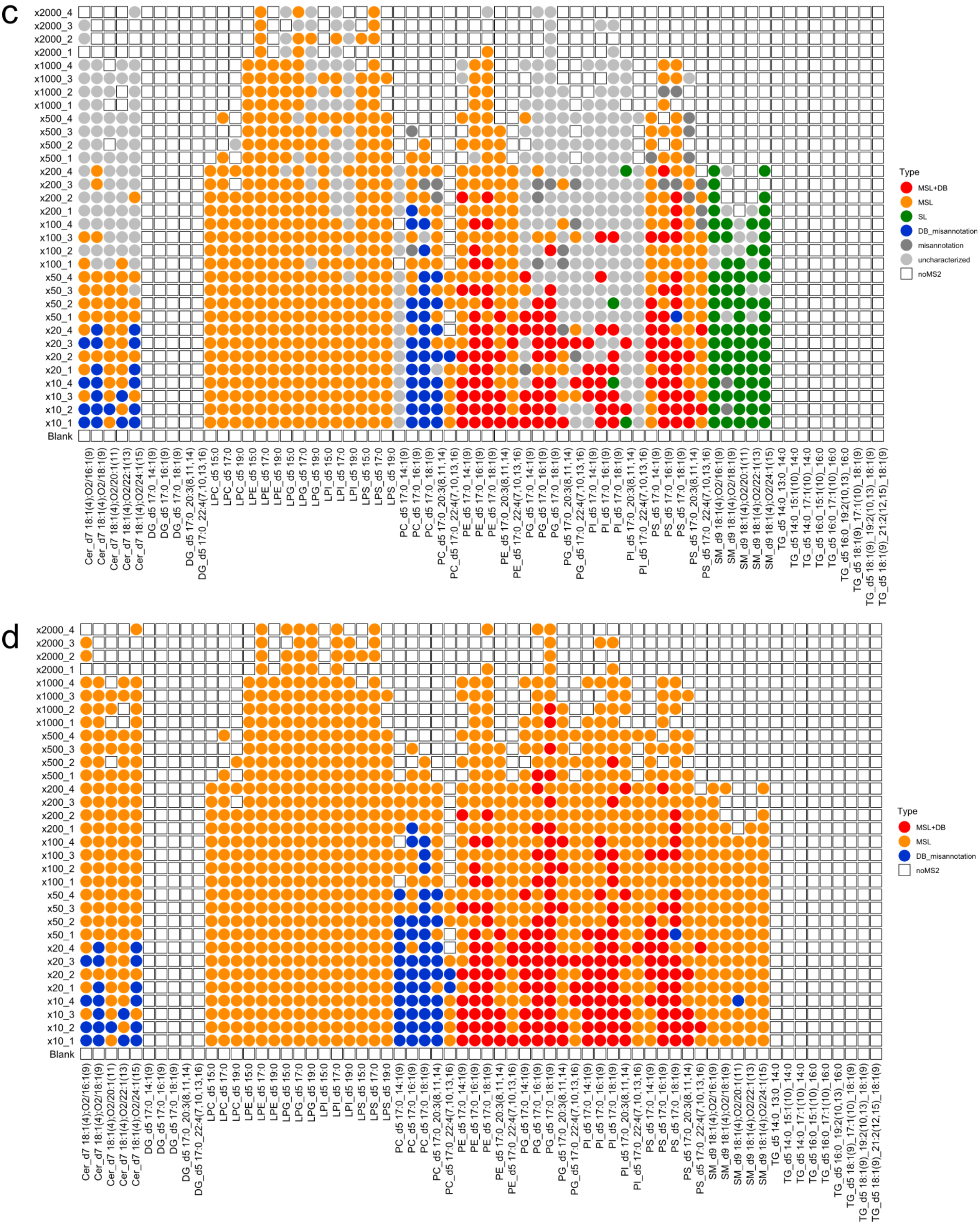

Figure S5. Annotated results of fatty acyl compositions and their C=C positions using MS-DIAL 5. Compared to Figure 2, the annotation results of individual samples were described. The definitions of color and symbol are the same as used in Figure 2c. (**a**) Results in positive ion mode. (**b**) Results in positive ion mode when the MSL annotations were carried out before the C=C positional annotations were performed. (**c**)

Results in negative ion mode. (**d**) Results in negative ion mode when the MSL annotations were carried out before the C=C positional annotations were performed.

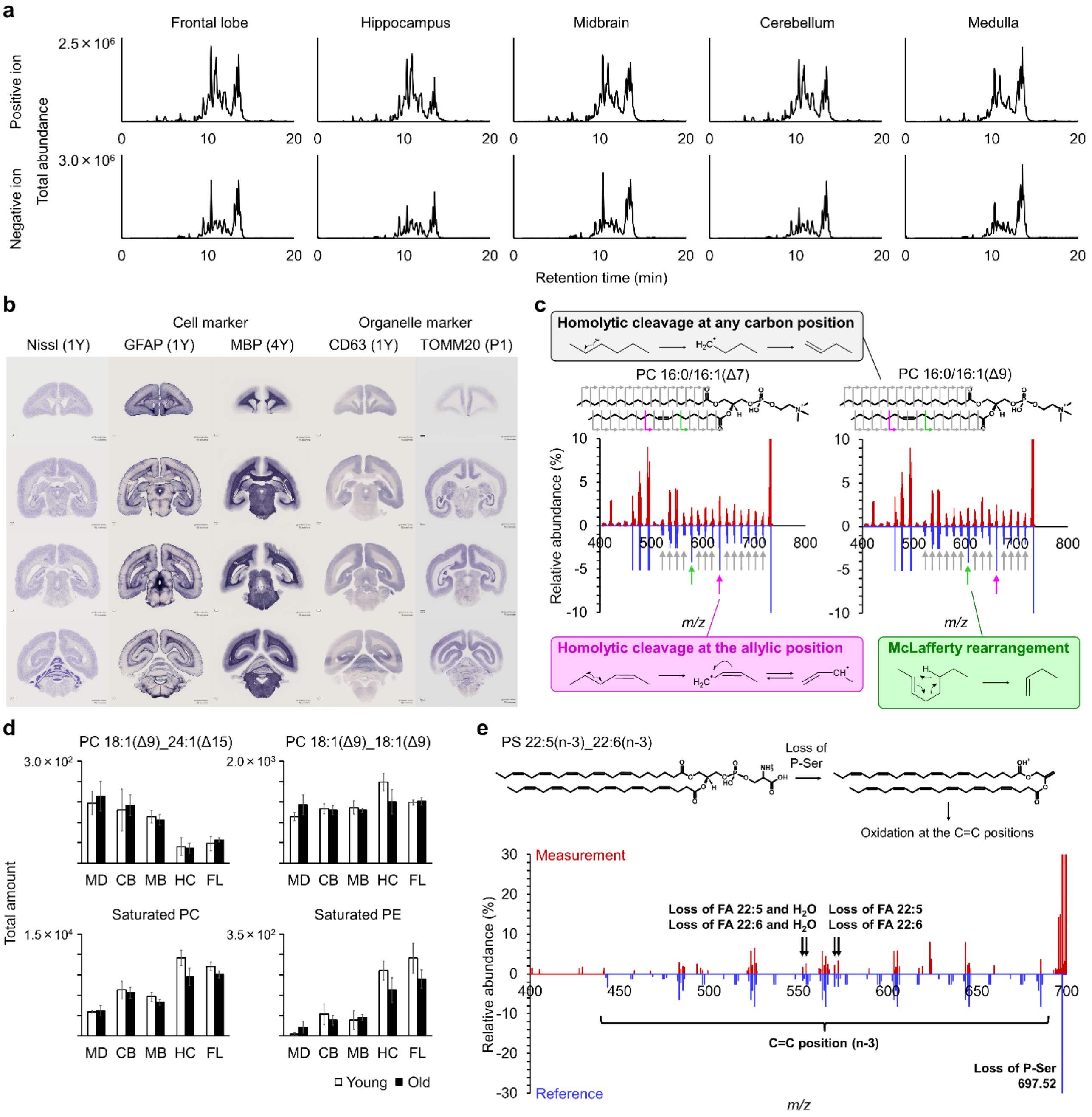

Figure S6. Double bond-resolved in-depth lipidomics of marmoset brain. (**a**) Total ion chromatogram of five sections of marmoset brain. (**b**) *In-situ* hybridization of cell and organelle markers in marmoset brain. The images were downloaded from the *Marmoset Gene Atlas* (https://gene-atlas.brainminds.jp/)[45,46]. (**c**) Structural analysis of PC 16:0_16:1 using EAD MS/MS. The peak of PC 16:0_16:1 at 10.1 min in Figure 4a was targeted. The C=C positions can be annotated with an allyl radical product and a hydrogen loss fragment produced by McLafferty rearrangement[22]. The lower blue mass spectra represent the reference library implemented in the MS-DIAL 5 software[40]. (**d**) Total amounts of *n*-9 MUFA-containing PC and saturated PC and PE in each region and age. Error bars indicate the standard deviations of the four biological replicates in each group. (**e**) Structural analysis of PS 22:5_22:6 using OAciD MS/MS.

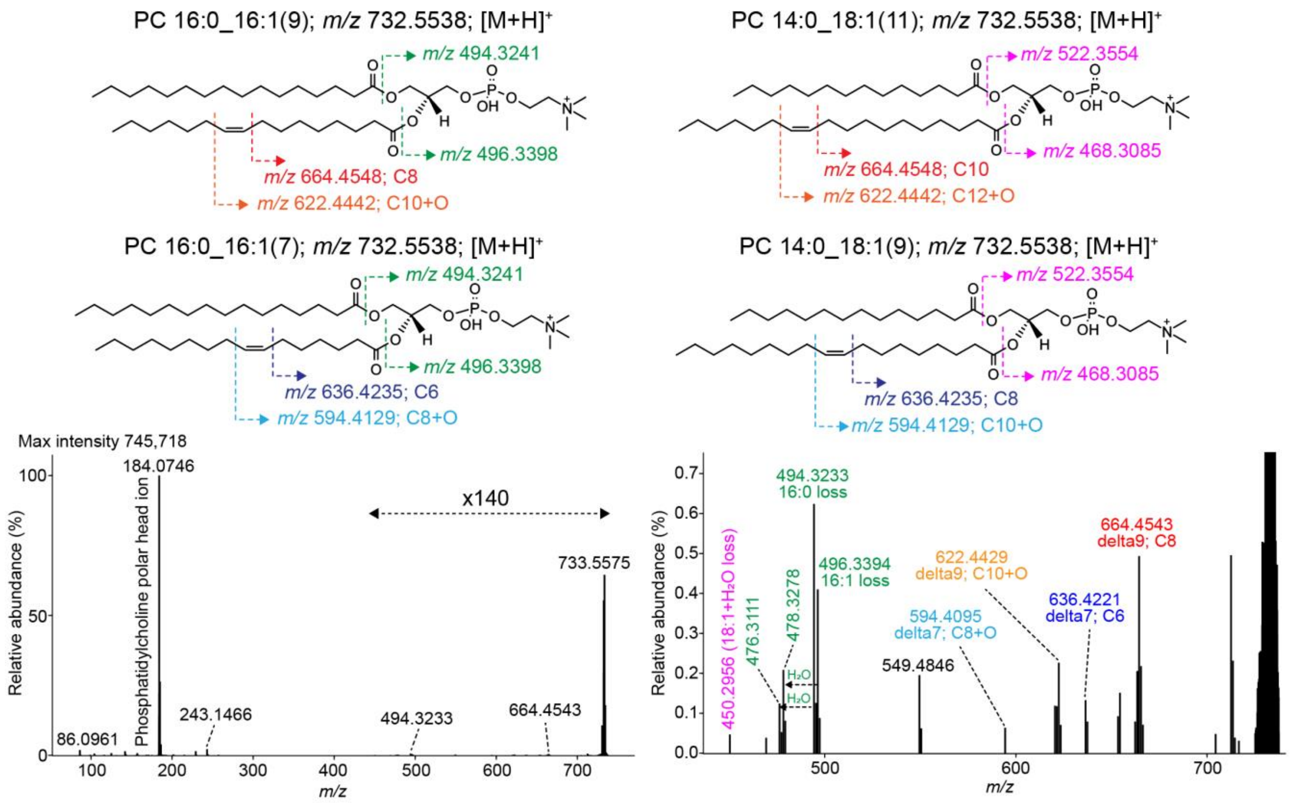

Figure S7. OAciD MS/MS spectra of the *m/z* 745.718 precursor ion and corresponding fragment interpretations. Four possible structural isomers, including PC 16:0_16:1(Δ9), PC 16:0_16:1(Δ7), PC 14:0_18:1(Δ9), and PC 14:0_18:1(Δ11), are described, all of which could potentially co-elute within the same retention time region. The diagnostic fragment ions used to determine acyl chain composition and C=C positions are also described for each isomer. The experimental MS/MS spectrum from a marmoset sample (file name: 20240130_Marmo9_young_hippocampus_OAD_POS_1_003), which exhibited the highest peak intensity among all samples, is shown. The left panel shows the entire MS/MS spectrum, while the right panel presents a zoomed-in view of the *m/z* 450 to *m/z* 735 region. According to the experimental spectrum, the contribution of PC 14:0_18:1 isomers is negligible, indicating that the diagnostic fragment ions for the C=C positions primarily originate from PC 16:0_16:1(Δ9) and PC 16:0_16:1(Δ7) (*m/z* 594 and 636 for *Δ*7, and *m/z* 622 and 664 for *Δ*9). No other structurally relevant fragment ions were detected, supporting the reliability of using *m/z* 664 and 636 to estimate the relative abundance of these isomers.
