# Supplementary material for: Dual fragmentation via collision-induced and oxygen attachment dissociations using water and its radicals for C=C position-resolved lipidomics
Source: Commun Chem. 2025 May 13;8:148. doi: 10.1038/s42004-025-01525-y (PMC12075507; doi:10.1038/s42004-025-01525-y)
Supplement: Supplementary file 3 — Description of Additional Supplementary Files [file 42004_2025_1525_MOESM3_ESM.docx]

**Description of Additional Supplementary Files**

**Supplementary Data 1.**

Structural analysis of lipid synthetic standards using sodium adducts. Each sheet summarizes system repeatability at different flow rates and compares sensitivity for each adduct ion. A product ion scan of deuterium-labeled EquiSPLASH standards is also included.

**Supplementary Data 2.**

Comparison of fragmentation patterns between Ar gas and H_2_O vapor with increasing collision energy. A product ion scan of deuterium-labeled EquiSPLASH standards is also summarized.

**Supplementary Data 3.**

Calibration curve of the RPLC/OAciD-MS system using the EquiSPLASH standard.

**Supplementary Data 4.**

Double bond-resolved in-depth lipidomics of marmoset brain. Lipidomics data were normalized using the sample volume (approximately 5 mg) and ion abundance of the internal standard added after lipid extraction by the modified Bligh and Dyer method. Detailed sample information (e.g., individual ID, sex, age, and analysis volume), lipid subclasses and their adduct ions used for lipid estimation, and MS-DIAL software parameters are also summarized.
